# Ewald Hering’s (1899) On the Limits of Visual Acuity: A Translation and Commentary: With a Supplement on Alfred Volkmann’s (1863) Physiological Investigations in the Field of Optics

**DOI:** 10.1177/2041669518763675

**Published:** 2018-06-04

**Authors:** Hans Strasburger, Jörg Huber, David Rose

**Affiliations:** Department of Medical Psychology and Medical Sociology, Georg-August-Universität Göttingen, Germany; Institute of Medical Psychology, Ludwig-Maximilians-Universität, Munich, Germany; School of Health Sciences, University of Brighton, East Sussex, UK; School of Psychology, University of Surrey, Guildford, Surrey, UK

**Keywords:** Hering, hyperacuity, visual acuity, eye movements, history of perception, Volkmann, Wülfing, Helmholtz, Vernier acuity, local sign, thresholds, psychophysics, German psychology, stereoacuity, irradiation

## Abstract

Towards the end of the 19th Century, Hering and Helmholtz were arguing about the fineness of visual acuity. In a talk given in 1899, Hering finally established beyond reasonable doubt that humans can see spatial displacements smaller than the diameter of a foveal cone receptor, an ability we nowadays call ‘hyperacuity’ and still the topic of active research. Hering suggested that this ability is made manifest by averaging across the range of locations stimulated during miniature eye movements. However, this idea was made most clear only in a footnote to this (not well known) publication of his talk and so was missed by many subsequent workers. Accordingly, particularly towards the end of the 20th Century, Hering has commonly been mis-cited as having proposed in this paper that averaging occurs purely along the lengths of the edges in the image. Here, we present in translation what Hering actually said and why. In Supplementary Material, we additionally translate accounts of some background experiments by Volkmann (1863) that were cited by Hering.

## Introduction

Ewald Hering (1834–1918) was a medically trained German physiologist who studied in Leipzig and subsequently also did research in Vienna and Prague. He made many original contributions, including on binocularity (e.g., the Law of Visual Direction) and on eye movements (e.g., the Law of Equal Innervation), and a conceptual breakthrough with his opponent-colour theory (or opponent-processing theory) which Helmholtz had wrongly dismissed at the time.

Hering’s father was a pastor, and his religious and cultural roots are sometimes cited as one reason for his lifelong antagonism towards the more cosmopolitan Helmholtz (Turner, 1994, p. 56). Indeed, several digs at Helmholtz appear in the Translation below. Academically, Hering favoured biological and nativistic explanations for perceptual phenomena, whereas Helmholtz proposed psychological explanations that emphasised learned experience.

By 1899, Hering was back working in Leipzig, having been called there in 1895. In the talk translated below, he contradicts Helmholtz’s denial that we possess the ability to detect spatial differences that subtend on the retina distances smaller than the diameter of a cone receptor (i.e., what we nowadays call hyperacuity: Westheimer, 1975). By outlining experiments by Wülfing and Volkmann, as well as his own, he established the reality of our apparently paradoxical ability to detect spatial changes in the retinal image an order of magnitude smaller than a foveal receptor.^1^ Moreover, he provided a theory to explain the phenomenon.

This was based on his earlier development of a ‘local sign’ theory.^2^ Each retinal receptor was postulated to carry ‘space values’ (Raumwerthe) that signalled its distance from the fovea along orthogonal (rather Cartesian) x- and y-coordinate axes, as well as depth by way of interocular differences in location along naso-temporal axes. He called these coordinates ‘breadth values’ (Breitenwerthe), ‘height values’ (Höhenwerthe), and ‘depth values’ (Tiefenwerthe), respectively (Hering, 1861). He realised that for a Vernier-style offset to be detected when it was less than the magnitude of one intercone distance, some integration of information was necessary. His diagrams of the situation (reproduced in our Translation) suggest that since the lines or edges extend over several cone diameters, the natural way to solve the problem would be to average – somehow – over the local sign values of the cones along the lengths of the contours (e.g., for vertical contours, above and below the offset). This is indeed how this paper has been widely cited: as the source of this theory (e.g., Badcock & Westheimer, 1985; Horton, Fahle, Mulder & Trauzettel-Klosinski, 2017; Matin, 1972; Levi & Waugh, 1996; Watt & Morgan, 1983; Westheimer, 2016; Westheimer & McKee, 1977).^3^ In consequence, some have claimed that Hering’s theory has been disproved because dot stimuli can exhibit hyperacuity (e.g., Ludvigh, 1953; Westheimer & McKee, 1977), and so too can curved line stimuli (Matin, 1972) – although in its defence, many others have shown length summation in hyperacuity experiments (e.g., Averill & Weymouth, 1925; French, 1920), if with qualifications (Wang & Levi, 1994).

However, although one could argue such averaging (along contour length) might be implicit in his theory, in fact Hering only proposed in this 1899 paper that *averaging happens over time, as ‘the incessant small movements of the eyes’ shift the image back and forth over the retina*. Perhaps this fact has been overlooked by subsequent workers because Hering gave the key part of the explanation in a footnote: designated footnote (d) in our Translation. In this footnote, he first revealed more about his underlying philosophy, saying that the mind creates idealised spatial structures, such as exactly straight lines, rather than merely recreating the retinal image (although by this he actually meant recreating the spatial pattern of retinal elements that are activated by the image). Otherwise, he said, a straight line would generally appear ‘gezahnt’ (literally, ‘toothed’; after some discussion, we have translated this here as ‘serrated’).

**Figure 1 and 2.**
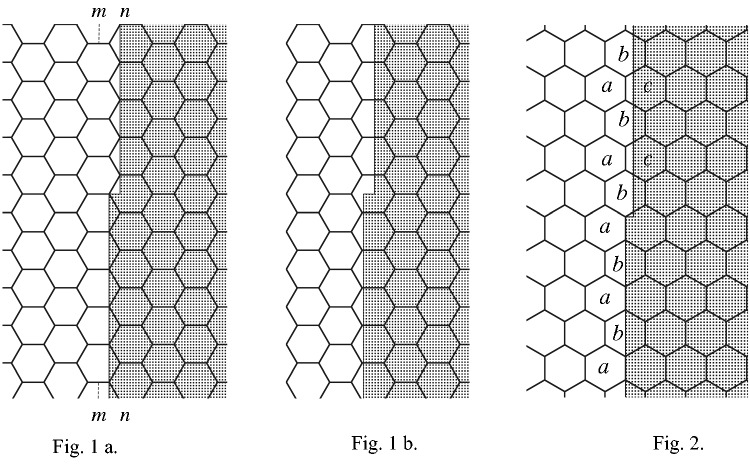

To understand this view fully, we need to consider first his figures in the main text. In these, Hering illustrates how he thinks straight edges in the image are perceived and how small offsets along their length are detected. For example, he assumed an edge that falls along purely a single line of receptors (such as the vertical set in Figure 1(b) in his paper) would be seen as being straight, despite the small offset (less than the diameter of a receptor) that occurs half-way along the edge. In contrast, the offset would be noticed when the eyes move the image slightly so that the two offset parts of the edge lie along different lines of receptors, even if only partially (as illustrated in Figure 1(a) in his paper). Thus, as the eyes move the image back and forth between the two situations (shown in Figures 1(a) and (b)), there will be a ‘*temporary* but repetitive detectability of the location difference’ (our italics) of the edge parts.

Behind this is a further assumption that the local signs (space values) attached to each receptor are *all-or-none*. Thus, the edge-parts above and below the offset in Figure 1(b) appear to be at the same (in this case, horizontal) location despite their different relative locations within the hexagonal receptors above and below the offset. It makes no difference to the perceived location of the edge just so long as each receptor receives an amount of light sufficient to make its sign ‘noticeable’ to the mind. The intensity of the light falling on a receptor is *not* indicated to the mind by the local sign.^4^ Hering believed this because, according to Lotze’s (1852) original theory, the local signs must be nonspatial (to solve the conundrum posed by Descartes’ reasoning that, because the mind is nonspatial, the signals sent to it cannot have any spatial quality). Thus, importantly, a local sign does *not* carry information about the size or diameter of the receptor element but merely signals its location, treated as a single point.^5^ Therefore, the number of receptors activated along an edge in the image does not matter, as long as these receptors have the same values along the relevant axis (i.e., breadth values for a vertical edge, as in Figure 1). Hence, length summation along the orthogonal axis would not make any difference, but merely replicate redundantly the information the mind is already receiving from the sign of, possibly, only a single receptor. In sum, Hering’s eye-movement hypothesis ingeniously made his theoretical stance and the empirical evidence consistent with each other, whereas *a length-summation explanation would in fact have been inconsistent with his theory*.

Subsequently, Andersen and Weymouth (1923) independently reinvented the theory of averaging across eye movements, and reproduced a figure just like Hering’s, which they had copied from Bourdon (1902, p. 146; note that although Bourdon had clearly read Hering’s 1899 paper, he did not mention Hering’s eye movement explanation). However, in 1925 Averill and Weymouth gave full credit to Hering as the originator of the idea in his 1899 paper (indeed they quoted from the relevant footnote – although only in German). They argued, however, that this is only one factor at work – alongside length summation and binocular summation – in improving acuity by (somehow) calculating a *mean* local sign. They provided empirical evidence for each factor, for example, testing the relevance of eye movements by varying stimulus exposure time.

But as explained above, subsequent writers in English have almost universally taken Averill and Weymouth’s (1925) passing of credit to Hering (1899) to refer to the length summation theory instead of to the eye movement theory or to a combination of these theories. In addition, the role of eye movements in hyperacuity has frequently been denied, for example, on the grounds that image stabilisation on the retina does not preclude hyperacuity (reviewed by Matin, 1972, p. 337; Rolfs, 2009, pp. 2429–2430; Steinman & Levinson, 1990, pp. 136–154), implying that ‘static’ properties of the eye determine the limits of acuity.

In fact, the relative popularities of these rival theories on the limits of acuity – those based on static (anatomical) or on dynamic (eye movement) factors – swung back and forth during the 20th Century (Rolfs, 2009; Steinman & Levinson, 1990). Most recently, however, it seems clear the pendulum has swung towards the dynamic. With more sensitive techniques, miniature eye movements during fixation have indeed been shown to be important factors in hyperacuity (Jiang et al., 2017; Rucci, Iovin, Poletti & Santini, 2007; Rucci & Victor, 2015) – as well as in other processes such as the prevention of perceptual fading even in the fovea (e.g., Costela, McCamy, Macknik, Otero-Millan & Martinez-Conde, 2013) and in acuity more generally (Ratnam, Domdei, Harmening & Roorda, 2017).

So Hering’s 1899 paper still bears relevance for modern perception research, both in establishing the existence of hyperacuity and as the first to suggest a positive role for miniature eye movements.^6^ It has been referred to in research across a wide range of topics over the more than a hundred years since its publication (even though its actual content may have evaded many). These topics include peripheral vision (Jüttner & Renschler, 1996; Levi & Klein, 1990; Strasburger, Harvey, & Rentschler, 1991; Westheimer, 1982), stereo vision (McKee, 1983; Westheimer & McKee, 1979), acuity in animals (Backhaus, 1959), and retinal implants (Eckmiller, Neumann & Baruth, 2005). Thus, the paper we translate here has played, and continues to play, a pivotal role in stimulating discussion of the most fundamental aspects of vision – how it is a spatial sense at all and how it is so admirably good at it.^7^

## Translation^8^

**E. Hering:** *On the Limits of Visual Acuity.* (Presented at the meeting on 4 December 1899. Manuscript submitted on 13 January 1900)

Following a presentation by Mr. Pulvrich on the C. Zeiss Co. stereoscopic distance meter, a discussion took place at the last convention of German natural scientists in Munich at which it was emphasised, by Mr. Lummer in particular, that a striking contradiction exists between the limits for the resolution of the eye, given amongst others also by Helmholtz, and the accuracy of binocular depth perception given by Mr. Pulvrich. The discussion did not achieve resolution of this contradiction. The contradiction exists, however, only as long as we overlook that something basically different is measured with the method commonly employed (including by Helmholtz) to measure visual acuity, compared to the determination of the accuracy of binocular depth perception.

One has become accustomed to use the visual angle of the smallest mutual distance at which two smallest possible points or narrow lines are just distinguishable as a measure of visual acuity. For example, double stars or line grids offered themselves as appropriate objects for such measurements, which can just about be resolved after as complete as possible accommodation. Yet, in this way, one determines the limits of optical resolution and not the actual fineness of the optical spatial sense, that is, one does not measure the smallest difference in position or size which the eye is just capable of recognising.

This difference is of fundamental importance, albeit not discussed anywhere as far as I know. I myself have been discussing it in my lectures for several years but have not found an opportunity to come back to it in public.

We should not, offhand, use the smallest distance between two finest points or narrow lines that are just resolvable as a measure of the fineness of the optical spatial sense. This follows first of all from the fact that, for example, the resolution of a bright double line presupposes the perception of a dark line in between, separating the two bright lines. Thence, one here recognises not only a difference in position of the two bright lines but at the same time the even smaller difference in position of the dark in-between line and either of the bright lines. Thus, the visual angle of the smallest difference in location perceived here does not correspond to the distance of the two bright lines, but corresponds to the location difference between the dark space in between and one of the bright lines. For the latter visual angle, however, only half of the first angle should be assumed at most. Thus, when, for example, a visual angle of 50′′ is found for the distance of a pair of lines that is just resolvable, the visual angle of the smallest recognisable difference in position is to be set at a maximum of 25′′.

It is, however, well established that the eye can even recognise much smaller differences in position. As early as 1863, Volkmann^a^ showed in his investigations ‘About the smallest relative size difference that we are capable of perceiving’ that – to mention just one example (p. 130) – two [horizontal] distances^9^ located next to one another – demarcated by the finest [vertical] wires – of initially 0.5 mm, or 0.9 mm or 1.3 mm, were confidently distinguished at 200 mm viewing distance when one of them was enlarged or diminished by $\frac{1}{90}$ mm, which corresponds to a visual angle difference of 12.4 seconds.^10,b^ Volkmann’s investigations on the limits of *un*noticeable differences (*ver*kennbare Unterschiede) of small magnitudes also led to values of the corresponding visual angles that extended *well* below 1 minute of arc.^11^

In the year 1892, Wülfing^c^ showed that one can recognise differences in position that correspond to a visual angle of 12–10′′ or even less. On a nonius-type apparatus, he shifted one half of a fine straight line against the other half by means of a micrometer screw until the position difference of the two halves became just reliably noticeable, and calculated the visual angle corresponding to the shift.

Wülfing also found his results in contradiction to the prevailing view and inferred from his experiments, which incidentally resulted in only a fifth of the value for the ‘smallest visual angle’ obtained by the double-object method, that the diameter of the retinal elements at the position of direct vision (respectively, the axial distance between two neighbouring retinal cones) must also be correspondingly smaller than hitherto assumed. This conclusion, which must strike us as odd considering the findings of histologists, is as we shall see directly, no more compelling than those drawn at the time by Volkmann, taking into account the effects of irradiation, from the results of the double-object method.

Let us, in the usual way, conceive of the retina’s central part as being divided into as many hexagonal area elements as there are retinal cones in the same area, and let us further assume that, for spatial perception, a space value (Raumwerth) goes with each of these visual field elements, as I will call them,^12^ that is just noticeably different from the space values of all of its neighbours. For a luminous double point to be still resolvable under such circumstances, the two retinal images or their respective irradiation areas must not get so close, or overlap so far, that a noticeably less illuminated visual field element does not still have space between the two illuminated visual field elements. The mutual distance of the points can therefore, even when we assume an (in reality never achievable) ideal acuity of their retinal images, never be smaller than the diameter of a visual field element. The same holds for double lines – by which I, including in the following, entirely ignore that the retinal image of a straight line, even with the most regular arrangement of the visual field elements, could meet an aligned flight of elements only in very special cases and in general falls on a more or less zigzag-shaped line of elements.^d^

While, therefore, an ultimate limit appears to be given in principle for the distance of resolvable double objects by the size of the visual field elements, the same does not hold true for spatial differences obtained with the Nonius method or the method of distance comparisons.

Let a surface, half of which is black to one side, the other half white, be divided into an upper and a lower half by a cut that is horizontal and at right angles to the straight line delimiting the white and the black, and let the lower half be movable against the upper half by means of a micrometer screw. As long as both halves of the vertical line are aligned, we see a single straight line, the apparent position of which is determined by the space values (breadth values) of all the visual field elements on which the image of the line falls. Presupposing the ideal but perhaps never fully realised case where the concerned visual field elements are arranged in straight, and coincidentally parallel, rows to the boundary line’s image, there are first of all two possibilities, illustrated by Figure 1(a), (b), and Figure 2.^13^ Figure 1(a) shows us the lower half of the image of the boundary line of white, lying on the element row *m-m* such that a small shift, as already shown by the upper half of the line image, is sufficient to excite, apart from the already aroused elements, also elements of row *n-n* by the light of the surface’s white half. As soon now as the excitation of the latter elements becomes sufficiently strong to become noticeable, the location difference of the two line halves will also become noticeable, insofar as our assumption is correct that each two neighbouring visual field elements have just noticeably different location values. Admittedly, a small shift of the line image on the retina will be sufficient to put both line halves once again onto one and the same element row of consistently equal breadth values, as shown in Figure 1(b), but another small shift of the eye in the same or opposite direction will shift both line halves once again onto rows of different breadth values, and in this way the, admittedly temporary but repetitive, detectability of the location difference can be sufficient to ensure the offset is perceived.

A second schematic case is depicted in Figure 2, where the boundary line coincidentally lies parallel to two sides of the regular hexagonal visual field elements. The lower half of the border line’s image runs, in turn, on the middle line of an element (*b*) and over the boundary line between two elements each; its apparent breadth location will therefore be the resultant of the breadth locations of the elements named *a* and *b*. The upper half of the white’s boundary has, however, shifted somewhat over the boundary of the elements marked a, to the right onto the element row indicated by *c*, and its apparent breadth location is determined by the breadth values of the elements marked *b* and *c*, and will be just noticeably different from the position of the lower image half, provided that the excitation of the *c*-elements becomes noticeable.

Those just described are borderline cases; to them could be added those in which the boundary line between black and white is at an arbitrary oblique angle relative to the row elements, and finally one could assume an arrangement of the visual field elements deviating more or less from the regular pattern. Always, however, one arrives at the result that under favourable conditions even the shift of one line-half by a fraction of an element diameter appears sufficient for just noticing the location shift – as long as the ‘light area’ of the retinal image corresponding to the white object surface declines sufficiently steeply at its boundary.

This is because the light emanating from a luminous point will not be reunited at a point on the retina even under the most favourable circumstances but illuminates a small area on the surface. If we imagine, for a given case, that in every point of such a small laminar point image an ordinate is raised whose height corresponds to the intensity of the illumination at its base, then we obtain the image’s light area (Lichtfläche), so-called by Mach. Likewise, the aforementioned boundary line of a luminous area in the exterior space does not lead to a sharp boundary on the retina, as we have assumed in our figures, but the light area reaches, with a more or less steep gradient, over the theoretically demanded boundary of the illuminated image part (physical irradiation). For the locational difference of the two halves of such a boundary line to become noticeable, if in the retinal image it amounts to only a fraction of one field element’s diameter, it is required that the aforementioned light area decreases sufficiently steeply to change noticeably the illumination of the newly excited row of elements with so small a shift. The value of the smallest noticeable locational change will thus be dependent likewise on the focus of the retinal image, the more or less favourable illumination intensity, and the adaptation state of the eye.

Analogous considerations to those just made also apply to the method of distance comparisons (Streckenvergleichung), as used by Volkmann.^14^ If one of the retinal images of the two adjacent distances is longer by only a fraction of an element diameter, it will, under favourable circumstances, affect one more visual field element than the other.

Binocular depth perception is another matter of perceiving positional differences, and analogous considerations apply to experiments about the accuracy of binocular depth perception as for the investigations using the Nonius method or the method of size comparison. Helmholtz has already attempted to measure this accuracy. He used three needles arranged initially in a fronto-parallel plane at 340 mm distance from the eyes, with a mutual distance of 12 mm. The central needle was then moved out of the plane of the other two, until its deviation from that plane became just noticeable, for which a displacement of ½ mm was sufficient. A binocular parallax of 60½ seconds of arc corresponds to the central needle at this deviation, if the two on the side are mapped onto corresponding [retinal] locations of the double eye (Doppelauge).^15^ This chance agreement with the smallest visual angle of a just resolvable double point or double line is the likely cause why Helmholtz did not vary his experiment at all, but concluded immediately ‘that the comparison of the retinal images of the two eyes for the purpose of stereoscopic vision occurs with the same accuracy with which the smallest distance is seen by one and the same eye’.^e^ This conclusion was unjustified, because here ‘the smallest distances seen by the eye’ meant the distances of the just still resolvable fine double objects and not the smallest still noticeable location difference (in breadth or height), and because a further variation of his experiment would have led Helmholtz, too, to entirely different values for the accuracy of depth perception.

Several years ago, Dr. Czapski of C. Zeiss Co. was so kind as to leave me two glass panes with engraved line systems that were made to study the limits of stereoscopic vision. On the pane I used, several groups of 5 mm long lines were found on either side (at [inter-]pupillary distance),^16^ the mutual distance of these lines being for the one eye fairly exactly 1 mm and for the other larger or smaller for some of the lines by small fractions of a millimetre. Since I am moderately short-sighted, I was easily capable – at viewing distances between 30 and 40 mm – to binocularly fuse two of these groups of lines each, under free viewing, and decide which lines appeared definitely closer or further away than their neighbours. The respective distance of the glass pane from the nodal point of the eyes was determined accurately, and the mutual distances of the lines were measured under the microscope. It emerged that under favourable illumination disparities of the lines corresponding to a visual angle of 11′′ effected a still confidently noticeable depth difference in the fused image. Imagine, thus, three 5 mm high vertical lines on each side, each separated by 1 mm from the other, and then the central line of the one group shifted sideways such that the distance of the shift corresponds to a visual angle of only 11 seconds: Then, this positional difference discloses itself in the binocularly fused images by a shift of the central line out of the plane of the other two. I did not test whether even smaller positional shifts of the lines would make themselves stereoscopically noticeable. Dr. Hofmann, who at my instigation made observations on the second pane, found a *threshold* of 11–12′′ visual angle. According to Pulvrich’s data, younger persons with fairly acute and continuously practised eyes are capable of recognising parallactic direction differences of 10′′ and even less as depth differences under free viewing. Nothing is reported about the objects used for these measurements; therefore, for the time being, a comparison with the experiments by Volkmann, Wülfing, and so forth is not possible.

In general, I will not address here at all the interesting and still insufficiently investigated relationships between the fineness of binocular depth vision and the fineness of binocular vision with respect to breadth and height, in short of depth-perception acuity and of area-perception acuity (Flächensehschärfe). Only one thing should be briefly mentioned, namely, that doing my observations using the line groupings on the above-mentioned glass panes, I observed again how much easier and more confidently small differences of two distances can be identified by using stereoscopic methods than by ordinary binocular observation, a fact that appears to me worthy of thorough investigation. For this, both observation methods should be applied comparatively using exactly the same objects.

Finally, it is barely worth saying that if one replaces the assumptions underlying this treatise about the field elements and their spatial relations to the retinal cones by considerably different assumptions, for example, by taking into account the difference of the cross-sections of the peripheral members to that of the central [foveal] members, then the interpretation of the discussed facts has to be different in part. Here, my aim was solely to demonstrate how these facts can be subsumed under the now common assumptions about visual field elements, whose correctness remains to be universally established.

## Supplemental Material

Supplemental material for Ewald Hering’s (1899) On the Limits of Visual Acuity: A Translation and Commentary Click here for additional data file. Supplemental material for Ewald Hering’s (1899) On the Limits of Visual Acuity: A Translation and Commentary by Hans Strasburger, Jörg Huber and David Rose in i-Perception

## Supplementary Material

Supplementary material for this article – an excerpt from Volkmann (1863) – is available online at: http://journals.sagepub.com/doi/suppl/10.1177/2041669518763675

## Notes to the Original Paper

- *Physiologische Untersuchungen im Gebiete der Optik I. Heft* [Physiological Investigations in the Field of Optics], I. Heft, Leipzig, 1863.
- For such small distances as were used here, the law of constancy of relative differences of just noticeably different distances no longer applies.
- Ueber den kleinsten Gesichtswinkel (On the smallest visual angle). Zeitschrift für Biologie, N. Folge. XI. Bd. S. 199.
- The spatial structures that develop in our consciousness on the basis of retinal images or, put differently, that are created by our faculty of imagination, are always schematic and idealised in comparison to the respective retinal image; otherwise an exactly straight line or an accurate circular line etc. could not exist at all amongst our visual percepts. If the image of a straight line on the retina were completely stationary, it could be asked why that same line does not generally appear to us serrated (gezahnt), corresponding to the locations of the activated retinal elements; since, however, as a consequence of the incessant small movements of the eye the line image constantly shifts on the retina, the relative space values of the individual line elements fluctuate within certain narrow limits around an average value, that latter being the determining factor for perception. Such considerations are inevitable, as long as one is prepared to accept such relatively large units, as the cones are, as visual field elements in the above derived sense.
- Handbuch der Physiologischen Optik (Handbook of Physiological Optics), I. Auflage, S. 645.

## Notes

1 In earlier days, the sizes of retinal receptors (assumed to be optic nerve terminals, since cones and rods were not discovered histologically until the mid-19th Century) had been estimated from psychophysical measures of acuity (Wade, 2004). Such measures of acuity, and estimates of receptor diameter based upon them as well as on histology, were well established at the time of Hering’s writings and accord with modern estimates of cone diameter (Wade, 1998, 2004, Table 1). However, these measures of acuity were also known to depend on the stimulus configuration, and could sometimes reveal perceptual limits much smaller than 1 min of arc, rather corresponding to modern hyperacuity values (Volkmann, 1863; Wade, 2004, pp. 882–883).

2 Hering’s theory has since been described both as ‘infamous’ (Turner, 1994, p. 66) and ‘an ingenious extension of the doctrine of local signs’ (Turner, 1994, p. 64). The latter refers to the theory of ‘local signs’ (Localzeichen) introduced by Lotze (1852); see Rose (1999) for further details.

3 Rare recent exceptions include Eckmiller et al. (2005) and Rucci et al. (2007).

4 Thus, ‘The local sign, or *Localzeichen*, is that which gives evidence of the spatial origin of a nerve signal. It is in the form of a hypothetical “extra process” (*Mitempfindung*) that accompanies the sensory message and gives a clue to its origin.’ (Morgan, 1977, p. 141); and ‘The local sign approach […] posits a second kind of information which is primarily concerned with where something is.’ (Braddick, 1993, p. 344). In other words, the intensity signal and the location sign are carried in parallel into the brain; the former varies parametrically with stimulus intensity whereas the latter does not: Each receptor just sends the one breadth value – or it doesn’t.

5 In modern terms, the activated cones act as point samples of the image. Each point is (somehow) coded as a triple of ‘values’ in three dimensions, according to Hering’s variant of local sign theory. Compare this with Helmholtz’s vision in which the filled diameters of active receptors do appear in perception, as is apparent in the diagram of hexagonally arranged receptors that he drew to explain how straight narrow parallel lines in fact *do* appear wavy (Wade, 2004, Figure 8) – a view that is even more Cartesian than Hering’s. However, Hering ignores all this here.

6 Before Hering, small eye movements during fixation had been assumed to degrade perception of detail. Jurin (1738), for example, proposed that eye movements might be the cause of the degraded recognition of ‘compound’ (multi-part) objects within what would nowadays be called a crowded display (Strasburger & Wade, 2015): ‘§175. […] the difficulty of keeping the eye perfectly steddy. […] by any imperceptible motion of the body, or involuntary fluctuation of the eye itself … From the same cause of the instability of the eye it must be, ceteris paribus, more difficult to perceive and distinguish the parts of any compound object, when each of those parts subtends a very small angle, than to see a single object of the same magnitude as one of those parts.’ (Jurin, 1738, pp. 150–151).

7 The subsequent influence and fate of Hering’s general theory of local signs is a different matter, which has been chronicled elsewhere (e.g., Matin, 1972; Rose, 1999).

8 We have tried to be as faithful to the original text as possible, but where ambiguity existed we have translated more freely to enhance comprehensibility. Occasionally we have added words [in square brackets]. Hering’s original footnotes have been renumbered and collated together at the end. They are indicated in the text with superscript lowercase letters. Our added footnotes are indicated by superscript numbers. Hering’s citations are given as originally in his footnotes and in full in our reference list. The original works of Helmholtz (1867), Volkmann (1863), Wülfing (1892) and Wundt (1874) were retrieved from the Internet Archive (https://archive.org/).

9 The word ‘Strecke’, translated here as ‘distance’, is used by Hering in a general mathematical sense as a (one-dimensional) spatial extent. In this example it refers to the horizontal distance between thin vertical wires and is the magnitude that is to be judged by the observer.

10 Volkmann’s experimental setup comprised three thin vertical wire threads of 0.05 mm thickness and 10 mm length each, the leftmost and rightmost of which were adjustable in horizontal position by a micrometer screw. One of the interwire distances was the reference, and the other was varied by the observer; left and right sides were balanced between trials. The three reference values were those stated earlier. The resulting threshold angle of 12.4′′ was added by Hering and is obviously incorrect (since arctan((1/90)/200) = 11.5′). However, Volkmann’s value of 1/90 mm, derived as the mean over 240 observations, was also incorrect, perhaps by a slip of the pen, and should have been 1/70 mm instead (the values summed across trials to 3.402 mm, which, when divided by 240, is 1/70 mm). Conspicuously, the specific example stating 1/90 mm (Experiment 79 on p. 130) was already picked by Wundt (1874, p. 555, footnote 4) out of Volkmann’s extensive set of data on the same experiment, with data varying considerably between subjects; so that might have been the reason why Hering chose it. As an aside, the correct value from the specific example in Volkmann’s data would have been 14.6′′. Wundt’s footnote 4 on p. 555 reads: ‘Fechner (elsewhere, p. 234) found $\frac{1}{40}$, Krause (in Volkmann, p. 130), at 200 mm viewing distance and 0.5–1.3 mm distance [found] $\frac{1}{90}$.’ – (‘FECHNER (a. a. O. I, S. 234) fand $\frac{1}{40}$, KRAUSE (bei VOLKMANN, S. 130) bei 200 Mm. Sehweite und 0,5–1,3 Mm. Distanz $\frac{1}{90}$.’) It occurs in a discussion of the Weber fraction for the visual-spatial sense (Augenmaass), so the cited fraction ‘$\frac{1}{90}$’ refers to Volkmann’s Weber fraction on p. 131 for the case of 1.3 mm distance, not the critical distance $\frac{1}{90}$ mm on p. 130, which Volkmann says is the same for the three distances. However, Wundt concludes on that basis that Weber’s law holds in that case, whereas Volkmann’s data on p. 131 shows it does not. Wundt must have been confused by Volkmann’s calculation error in the same way that Hering was later.

11 Volkmann introduces the term ‘verkennbare Unterschiede’ (as opposed to ‘erkennbare Unterschiede’: noticeable differences) in §54 on page 96, after the description of Experiment 59 (in which he reports a just noticeable difference that is ‘by 22 times smaller than the smallest cone diameter’). For the definition, Volkmann takes the example of a line-thickness comparison task, and in a statistical aside on the next page he further explains that, even though the *verkennbare* differences will be smaller in the mean than the *noticeable* differences, their maximum can be larger than the minimum of the latter. What he means by ‘verkennbar’ becomes clear only much later in the book, however, on page 118 (in §65). In that section he refers to Fechner’s just noticeable difference (jnd) and its role in Weber’s law, and introduces a counterpart of a just *unnoticeable* difference. That is, whereas a jnd is just above threshold, a just unnoticeable difference is just below threshold. Both obey Weber’s law: “Here one will, in general, commit a certain error that depends on [the fact] that the size differences are only perceivable up to a certain limit of smallness. To understand the significance of these errors correctly, one needs to take into account that an error, made in such equalization experiments, represents the size of a difference that went unnoticed. Considering now that the task of equalization is solved by decreasing a just noticeable difference until it transits into the unnoticeable, it is evident that, when proceeding carefully, the *un*noticeable (*ver*kennbare) difference will only be by a minimum smaller than the just noticeable. Thus, if the values of the smallest noticeable size differences [jnds] are, by Weber’s law, relative ones, i.e., with respect to the dimensions they are referenced to and increasing like them, then the smallest *un*noticeable differences must behave accordingly. They, too, must increase with the dimensions they are referenced to and, approximately, increase like those”. “From all this it follows that the validity of Weber’s law can be assessed not only by the just noticeable but also by the just unnoticeable size differences, i.e., by the *errors* made in equalization experiments”. “When experiments with the same normal distance are repeated sufficiently often, by division of the sums of errors by the number of observations, one obtains an error that is proportional to the mean value of the just *noticeable* difference and that is equal to the mean value of the *just unnoticeable* difference” (Volkmann, 1863, p. 117–118). Hering appears to not have read Volkmann’s treatise carefully, since the (wealth of) data provided by Volkmann on perceivable distances that are *far* below the cone diameter, have little or nothing to do with an analysis of the just *unnoticeable* difference!

12 ‘Sehfeldelemente’; this auxiliary term coined here by Hering is roughly synonymous with the retinal area covered by a receptor. The term ‘receptive field’ could be used but is typically applied only to neurons later in the pathway; the term is unrelated to the *visual field *(which would be *Gesichtsfeld)*.

13 Although thin black lines are drawn for clarity along the black-white borders here, as they are in Hering’s original figures, it is clear from his text that the borders are intended to be thought of as single step edges in luminance.

14 It is not clear whether this comment refers to the comparison of the two distances in the experiment described in the foregoing (where the term *Strecke* is somewhat uncommon for a distance), or to Volkmann’s Chapter VI ‘On single vision with two eyes’. The latter is where the word *Strecke* first appears in Volkmann’s book (on p. 246), and Hering changes the subject to stereoscopic vision in what follows in his essay. Volkmann, at the referred location, calculates the difference between two horizontal distances – the minimum and maximum distance in one eye between its fovea and a point *b* that corresponds to a point *B* in the other eye. That difference is not really a comparison (Vergleich), however, but rather denotes the range within which points are still stereoscopically fused, i.e., it denotes the horizontal extent of Panum’s area. Volkmann’s Chapter VI (pp. 181–268) is easily overlooked, by the way, because it is missing in the table of contents!

15 Hering had used the term *Doppelauge* (double eye) before (Hering, 1861, p. 20). Helmholtz (1867, pp. 611, 612) called Hering’s *double eye *the ‘cyclopean eye’, and this is the term in use today (see Wade, 1998, for the history of the cyclopean eye concept preceding Hering).

16 Hering gives few details of their spatial layout, but one possibility is that the groups (each of three lines) were arranged in left-right pairs so as to allow free fusing, with further pairs below, running down the pane.
