# Supplementary material for: Ewald Hering’s (1899) On the Limits of Visual Acuity: A Translation and Commentary: With a Supplement on Alfred Volkmann’s (1863) Physiological Investigations in the Field of Optics
Source: Iperception. 2018 Jun 4;9(3):2041669518763675. doi: 10.1177/2041669518763675 (PMC5990881; doi:10.1177/2041669518763675)
Supplement: Supplemental material for Ewald Hering’s (1899) On the Limits of Visual Acuity: A Translation and Commentary [file Supplemental_material.pdf]

Alfred Volkmann (1863)

Physiological Investigations in the Field of Optics (Physiologische Untersuchungen im Gebiete der Optik)

Leipzig: Breitkopf und Härtel

Translated by Hans Strasburger[1] and David Rose[2]

Alfred Wilhelm Volkmann (1801–1877) was a German physiologist, anatomist, and philosopher who worked in Leipzig, Dorpat (currently, Tartu, Estonia) and Halle. He performed original research on a range of topics including neuroanatomy and optics (e.g. 1836, 1846, 1863) which was frequently cited by Helmholtz (1867). In his 1863 book he reported extensive psychophysical studies on visual acuity.

In the following we translate accounts of some background experiments by Volkmann that were cited by Hering (1899). Our centre of interest is the result of Experiment 79 on p. 130, picked (somewhat arbitrarily and perhaps following Wundt, 1874) by Hering as an example for the just noticeable difference of distances in Volkmann's line-separation experiments. However, due to a little slip-up Volkmann reports the wrong value on p. 130, perhaps by a calculation error or by writing down one of the three Weber fractions from the following page ($^1/_{90}$) as the just noticeable distance difference of $^1/_{90}$ mm.

Hering (1899) further draws a connection to Volkmann's concept of a *just unnoticeable (verkennbar) difference*, and we therefore draw attention to §54 on p. 96, where Volkmann introduces the term, and to §65, p. 118, where it becomes clear that the term is meant as a counterpart to Fechner's *just noticeable difference*.

Hering finally refers to Volkmann's method of distance comparisons (Streckenvergleichung), and that appears to refer to Volkmann's Chapter VI "On single vision with two eyes" (pp. 181–268). There on p. 246 the term *Strecke* first appears in Volkmann's book. His Chapter VI could easily be overlooked because it is missing in his Table of Contents.

References

Helmholtz, H. (1867). *Handbuch der Physiologischen Optik, I. Auflage (Handbook of Physiological Optics, 1st Edition)*. Leipzig: Leopold Voss.

Hering, E. (1899). Ueber die Grenzen der Sehschärfe *(On the limits of visual acuity). Berichte über die Verhandlungen der Königlich-Sächsischen Gesellschaft der Wissenschaften zu Leipzig. Mathematisch-Physische Classe; Naturwissenschaftlicher Teil,* 51, 16–24.

Titchener, E. B. (1905). *Experimental Psychology, A Manual of Laboratory Practice, Vol. 2.* New York: Macmillan.

Volkmann, A. W. (1836). *Neue Beiträge zur Physiologie des Gesichtssinnes (New Contributions to the Physiology of the Sense of Sight).* Leipzig: Breitkopf und Härtel.

Volkmann, A. W. (1846). Sehen (Sight). In R. Wagner (Ed.) *Handwörterbuch der Physiologie, mit Rücksicht auf physiologische Pathologie (Concise Dictionary of Physiology, with Consideration of Physiological Pathology)* Dritter Band, Erste Abtheilung (Vol. 3 Part 1), pp. 265–351. Braunschweig: Vieweg.

Volkmann, A. W. (1863). *Physiologische Untersuchungen im Gebiete der Optik, I. Heft (Physiological Investigations in the Field of Optics, 1st issue).* Leipzig: Breitkopf und Härtel.

Wade, N. J. (2004). Visual neuroscience before the neuron. *Perception, 33,* 869–889.

Wade, N. J. (2010). Pioneers of eye movement research. *i-Perception, 1*, 33–68. dx.doi.org/10.1068/i0389.

Wundt, W. (1874). *Grundzüge der Physiologischen Psychologie, Vierzehntes Kapitel (Outlines of Physiological Psychology, Ch. 14).* Leipzig: Wilhelm Engelmann.

[1] Universities of Munich and Göttingen, Germany

[2] University of Surrey, UK

Table of Contents

Page

I. On irradiation 1

II. On the relationship between the intensity of a stimulus and the intensity of sensation (Empfindungsstärke) 51

III. Investigations on circles of sensation (Empfindungskreise) and isolated nervous conduction 65

IV. Whether the smallest relative size differences that we are capable of perceiving have a constant value 117

V. On what is primordial and acquired in spatial perception 139

[For some reason the Table of Contents stops here, even though the book has further chapters. From scrolling through the book, Chapter V ends on page 180, and on page 181 Ch. VI starts:]

VI. On single vision with two eyes. 181

End of Ch. VI and end of book 268

p. 1

I. On irradiation

§1. A brightly illuminated area on a dark background appears larger that it really is; it is enlarged at the cost of the darker surround. As is generally known, physics has summarized the pertinent phenomena under the term irradiation.

Whereas PLATEAU sought the cause for irradiation in physiological relationships, and assumed that the retina's excitation by a bright surface exceeds the limits of the optical image, WELCKER proved that its cause is physical and lies in the scattering of the light. This explains why irradiation increases with the inaccuracy of accommodation, and that it can be removed by suitable spectacle lenses but can also be elicited by unsuitable ones. […]

§2. Dark objects on light ground can also irradiate, i.e. can appear magnified at the expense of the bright surround. A simple means to convince oneself of that is the following. Draw on very fine, white paper a pair of equally delicate black lines in a way that they cross at an angle of 1 – 2°. View the drawing then at a suitable distance and note the point where the line thickness appears equal to their mutual distance.

p. 2

If one then inspects one's estimate by means of a strong magnifying glass, one finds that the distance between the lines is considerably larger than their thickness, an immediate proof that the black lines appear thicker than they are and have widened themselves on the expense of the white space in between.

p. 3 [bottom]

§5. My experiments were, in part, performed with two parallel silver wires of 0.05^{mm} thickness and 10^{mm} length. (Footnote: In my first treatise on irradiation I specified the wire

thickness as 0.0445^{mm}. In the mean over numerous measurements using four different lenses on an OBERHÄUSER microscope, the value given in the text is the more precise one.)

p. 4
Viewed against the bright sky these wires appear as dark lines and, by means of a very precise screw micrometer, can be moved without violating parallelism, i.e. brought nearer to one another, at will. The extent of their mutual distance which, according to the task, has to match the apparent line thickness can be measured down to 0.01^{mm}, and estimated down to 0.001^{mm}. In my above-mentioned first treatise I have also viewed the silver wires with incident light in front of a black background, by which I hoped to determine the given differences that characterize the irradiating of black and white objects. Even though by these experiments predominance on the side of the white objects was fully proven, the extent of the difference was not possible to fathom by my approach. The light reflected from the cylindrical wires brings about images that are inevitably slimmer than the wires themselves, and by this the wire's thickness that is to be employed in the calculations becomes questionable.

p. 65

III. Investigations on circles of sensation (Empfindungskreise) and isolated nervous conduction

§38. The spatial senses are idiosyncratic in that the stimulation of a sensible point *a* invariably evokes a different sensation than the stimulation of a sensible point *b*, namely one spatially different. This also happens when the stimuli that hit the two points are of equal composition. Let us consider a playing card, e.g. the Five of Diamonds [Carreau-Fünf], we distinguish the 5 quadrangles, even though they tally fully with regard to form, size, and colour, whereas with 5 equal tones that reach our ear simultaneously we cannot distinguish anything.

One may assume that this peculiarity of the spatial senses yields the fundamental condition of all spatial perception (räumliche Anschauungen). Only because the soul (Seele, the mind) is affected differently by each point of the sensible area, even if the stimulus should be qualitatively the same, is there reason to conceive the affections emanating from different points as the points being next to and yet apart from one another (… die von verschiedenen Punkten ausgehenden Affectionen in der Form des Nebeneinander und Aussereinander aufzufassen).

p. 66
Let us consider a white disk that, by two crossing lines, is divided into four quadrants, […]

p. 67
The first important fact to which WEBER has drawn attention is that pressure and thermal stimulation will be perceived by any skin location as of equal acuteness (Schärfe) whereas the distance of two simultaneously stimulated points will be perceived far more in fineness at certain skin locations than at others (Footnote on an objection by GOLTZ). This proves, in any case, that other facilities need to supervene those that mediate pressure and warmth sensations.

p. 69 [bottom]
[Explanation of the injury, needed for the next experiment:]

About five years ago I suffered a contusion at the distal phalanx of the right forefinger, and

the sensitivity of the fingertip, that seemed to have been lost for some time, did not return to its original acuteness even after the wound's healing. This blunting of sensitivity is based undeniably on part of the ruptured nerve fibres not having regenerated and there is thus a skin region available in which one can study how the percepts of size (Grössenanschauungen) turn out when the nerve fibres that signal the respective area's states of excitation to the centre [i.e. to the CNS] are partly destroyed.

p. 70

The experiments are pursued in the following way. Given are, firstly, two [dividers'] tips with fixed position at a constant distance of 2 millimetres, and secondly two movable tips whose distance can be varied by a screw at will and, by a suitable arrangement, can be measured to an accuracy of $^1/_{20}$ millimetre.

The experiment's task is to equalize the two distances by tactile sensation only.

Experiment 35

[Tactile sense. Introduction of the term *Fehldistanz*. This technical term introduced by Fechner, literally perhaps '*deviating distance'*, would nowadays be referred to as the *test distance* in a forced-choice comparison task. Titchener (1905) in "Experimental Psychology, A Manual of Laboratory Practice, Vol. 2" has left it untranslated.]

The distance of the fixed tips (normal distance) was sensed by the left forefinger, the distance of the moveable tips, however, by the injured right forefinger. Considering the sensory differences arising from one or the other [tactile] palpation, the variable distance was corrected until it appeared equal to the constant one. The values of the deviating distances (Fehldistanzen) (which, following Fechner, I will call those distances that emulate the normal distances) were the following:

p. 71

3.65 3.25 3.2 3.4 3.3 3.4 3.1 3.6 3.35 2.85^{mm}, in the mean over the ten experiments 3.25^{mm} [it should be 3.31; perhaps a rounding error]. When using the injured finger, a distance was thus brought about that was by $^1/_{13}$ too large, which can only be caused by him underestimating the fingered distance by $^1/_{13}$.

Experiment 36. [Tactile sense]

§40. Panum (Die scheinbare Grösse der Gesichtsobjecte; v. Gräfe Arch. f. B. V. S. I.) remarks: The assumption that the apparent size of the visual objects depends on the size of the stimulated circles of sensation […]

[Volkmann changes the regard here to the visual sense, starting with a comparison of the peripheral and central retina]

p. 78

[About Weber noting the extremely different acuity for central and peripheral vision and in §46 about Heinrich Müller having taught us that there are only cones in the fovea]

According to Kölliker, cone diameter is 0.0020 – 0.0030''',[3] or 0.0045 – 0.0067^{mm}; according to H. Müller: 0.0040 – 0.0060^{mm}. With these, the reports of Gerlach and Frey concur almost fully. Bergmann reports that his measurements led to the minimum values of Kölliker's, i.e.

[3] Kölliker, and on the next page Schultze, use the Paris line as the measure of length here. One Paris line, abbreviated as 1''', is one twelfth of a Paris inch (or pouce) and equals 2.2558 mm. Volkmann converts the results to the metric system (cf. Wade, 2004).

0.0045^{mm}.

p. 79
From letter messages by M. SCHULTZE, the cones in the yellow spot's centre are by about a half smaller than at its circumference and in the *fovea centralis* only measure 0.0010—0.0012''' or 0.0022—0.0027^{mm}. Importantly, H. MÜLLER confirms these reports from SCHULTZE in his latest work on the cones at the human yellow spot.

[…]

A. Inferences on the size of the retina's elementary parts from the size of the smallest visible distances

§47. On the values of the smallest visible distances there are numerous but only imperfect observations. Namely, the influence of irradiation has not been taken into account, which is significant and in any case not negligible. By irradiation of the observed lines or points, the distance between those has diminished on each side by the radius of a blur circle. No matter how minor the reduction in absolute terms may be, it is nevertheless a discernable one, and for the distances that lie on the border of the visible consequently an influential one.

p. 80
The screw micrometer described above (§5) served as instrumental means. In all experiments the instrument was set up such that the wires stood out as dark threads from the bright background. The eye's distance from the two wires was held constant in each experimental series and corresponded to the most suitable viewing distance.

To allow an exact understanding of the pursued procedure I will describe one experimental series in all detail, and for its abbreviation will introduce the following symbols:

S Most fitting viewing distance (Sehweite), measured from the object to the eye's mean nodal point.

D Smallest visible distance between the micrometer wires.

D' Distance of the micrometer wires that appears equal to the wire thickness

δ Smallest discernable retinal distance without accounting for irradiation

δ' Smallest visible retinal distance with accounting for irradiation i.e., after subtraction of the blur circle.

Z The widening of the wires by irradiation, which reduces their apparent distance [separation].

ζ Size of the blur circle on the retina, from which it parenthetically follows that $\delta' = \delta - \zeta$.

p. 81
Experiment 37 [performed by the author, description of the author's eye in Exp. 13]

[In the following an exemplary description of the calculations. Note that most researchers in those days used only one subject, on the assumption all (non-clinically defective) eyes were much the same (Wade, 2010, pp. 42-43). So we should give credit to Volkmann for using many different observers!]

p. 82
Experiment 38 [performed by the author, conditions as in Exp. 37]

Experiment 39 [performed by Prof. Vogel, conditions as in Exp. 37]

p. 83

Experiment 40 [performed by stud. Solger, conditions as in Exp. 37]

Experiment 41 [performed by Dr. Schweigger-Seidel, conditions as in Exp. 37]

Experiment 42 [performed by MScEng. (Ing.) Volkmann, conditions as in Exp. 37]

Experiment 43 [performed by Dr. A. Gräfe, conditions as in Exp. 37]

Experiment 44 [performed by stud. med. Geiss, conditions as in Exp. 37]

p. 84

Experiment 45 [performed by Mrs. A., conditions as in Exp. 37]

Experiment 46 [performed by Mrs. G., conditions as in Exp. 37]

Experiment 47 [performed by trainee (Referendar) Volkmann, conditions as in Exp. 37]

Experiment 48 [performed by MScEng. (Ing.) Theodor, conditions as in Exp. 37]

Experiment 49 [performed by Dr. Sievert, conditions as in Exp. 37]

Experiment 50 [performed by Mrs. A., conditions as in Exp. 37]

Experiment 51 [performed by Mrs. V. S., conditions as in Exp. 37]

Experiment 52 [performed by stud. med. Jahn, conditions as in Exp. 37]

Experiment 53 [performed by stud. med. Krause, conditions as in Exp. 37 in daylight and repeated in lamp light, "such that the wires set themselves apart as black threads from the lamp's white translucent glass"]

Experiment 54 [performed by Prof. Welcker] (p. 85)

p. 92, 93

Allow me to summarize the results of the experiments on the smallest noticeable distances in a few words. When the influence of irradiation is duly taken into account, the smallest noticeable distances are, even for the untrained observer, half as large as the retinal cones' diameter, with the latter's value assumed very low, at 0.0025^{mm}. Yet through training the smallest noticeable distances were [further] reduced by a factor of two, such that they correspond to only one fourth of a cone diameter. Now, since the perception of a between-space appears to depend on several, and namely at least two, sensory circles (Empfindungskreisen), it is not to be assumed [it is not likely] that the diameter of such a circle is larger than $^{1}/_{8}$ of a cone diameter.

p. 96

§54. The sizes of the sensitivity circles can also be deduced in yet another way from the smallest visible differences, and the considerations that bring themselves to bear are rather simple ones.

When one pursues the task of equalizing a magnitude *g* to another magnitude *g'*, and avails oneself only of visual judgement, one will rarely wholly succeed. Nearly without exception more or less substantial differences will reveal themselves at remeasurement of the two magnitudes, and it is evident that these are differences that the eye is not capable of perceiving. These sometimes larger sometimes smaller differences revealed by the equalization experiments (in other words the errors of equalization (Ausgleichungsfehler) thus teach us about the limits of *unnoticeable (verkennbare)* differences.

p. 97
I will denote these differences by *u′*, while I keep the letter *u* for the smallest noticeable *(erkennbare)* differences introduced in §52.

It is easily seen that the two magnitudes, *u′* as well as *u*, will vary within certain limits; because a difference that one notices in certain cases, one will not notice under other circumstances. Hence, although the *unnoticeable (verkennbare)* differences will generally be smaller than the *smallest noticeable (kleinste erkennbare)*, in large series of observations the opposite relation will emerge at times. From this it follows that the maximum errors in equalization experiments will lead to *u′* values that are not smaller but are larger than the *u*-values at their minimum.

[This seems to be a statistical aside, saying that even though *u′* will be smaller in the mean, its maximum can be larger than the minimum of *u*.]

The equalization experiments [described] in the following were performed with a micrometer screw that differed from that described in §5 only in that a third, fixed thread is attached between the two movable threads. These 3 threads delimit 2 distances, and thus, if the observer is presented with one distance (Fechner's normal distance), the other (Fechner's *Fehldistanz*) can be varied by the observer at will, and, in so far as visual judgement (Augenmaass) allows, can be made equal to the former. The thickness of the wire threads was again 0.05^{mm}, and the fineness of the measurements was also the same.

p. 97 [bottom]

Experiment 60

Performed by stud. med. GEISS. The distance of 0.3^{mm} is given. In 192 trials the maximum deviation of the deviating distance (Fehldistanz) was 0.012^{mm}.

p. 117

IV. Whether the smallest perceivable relative size differences have a constant value

§64. For all intensive stimuli the law appears to hold that the difference in sensation stays the same when the ratio of the stimuli [the stimulus intensities] stays the same. Following E. H. WEBER, this law also holds for extensive stimuli, so that the smallest noticeable size difference is given by a constant ratio of the two dimensions that are put in comparison.

FECHNER (Elemente der Psychophysik II, p. 343) has subjected to an extensive inquiry the question of whether the law that applies to intensive stimuli should be extended to extensive stimuli, and has proven that in the realm of the tactile sense at least the assertion must be rejected. In contrast, the corresponding experiments on the spatial sense (Augenmaass) nearly always obeyed WEBER'S Law. Only when the dimensions that were put in comparison were particularly small, peculiar exceptions occurred that, by a certain regularity (Gesetzlichkeit), pointed to a new, as yet unknown, cause for these phenomena.

Even though I cannot fully solve the various difficulties connected to the just mentioned issue, I still decided, following FECHNER'S advice, to compile material that has been collected at great expenditure of time. Indeed, already as presently available it opens up many a point of view, and in any case those experiments performed by myself are to be viewed as groundwork without which the relationship of the extensive stimuli to the extensive sensations could by no means be fathomed.

§ 65. The experiments were firstly performed by the method of mean errors. If a certain

distance (FECHNER'S normal distance) is given, a second distance (FECHNER'S *Fehldistanz)* is sought to be made equal to it.

p. 118

Here one will, in general, commit a certain error that depends on [the fact] that the size differences are only perceivable up to a certain limit of smallness. To understand the significance of these errors correctly, one needs to take into account that an error, made in such equalization experiments, represents the size of a difference that went unnoticed. Considering now that the task of equalization is solved by decreasing a just noticeable difference until it transits into the unnoticeable, it is evident that, when proceeding carefully, the unnoticeable (verkennbare) difference will only by a minimum be smaller than the just noticeable. Thus, if the values of the smallest noticeable size differences [jnd] are, by WEBER'S law, relative ones, i.e., with respect to the dimensions they are referenced to and increasing like them, then the smallest unnoticeable differences must behave accordingly. They, too, must increase with the dimensions they are referenced to and, approximately, increase like those. From all this it follows that the validity of Weber's law can be assessed not only by the just noticeable but also by the just unnoticeable size differences, i.e. by the *errors* made in equalization experiments.

When experiments with the same normal distance are repeated sufficiently often, by division of the sums of errors by the number of observations one obtains an error that is proportional to the mean value of the just *noticeable* difference and that is equal to the mean value of the *just unnoticeable* difference.

Concerning the experimental procedures, I use 3 parallel, in most cases vertical [wire] threads for the equalizations [adjustments], the mutual distances of which are variable at will. It will matter whether the reference distance is left or right of the observer, and therefore the deviating distance (Fehldistanz) is obtained by variation of the thread farthest to the right or farthest to the left. With respect to that it is required to alternate with the spatial location of the reference distance, and perform an equal number of experiments in the one and the other way.

p. 128

[Experiment 78, performed by stud. med. Krause]

	Distances in millim.					
	0.3	0.5	0.7	0.9	1.1	1.3
S	1.254	1.296	1.340	1.380	1.367	1.265

SS = 7.902   F = 1/118

The error sums in this last experiment are noticeably the same for all distances.

§68. […]

In the preceding I already specified the mean error of equalization (mittlere Ausgleichungsfehler) for each experimental series as a total, and denoted it by *F*. The aim now is to calculate the *relative* error of equalization for each distance that was used separately. For this, one divides for each singular distance the error sum *S* by the value of that distance, and further divides that ratio by the number of observations = $2m\mu$. […] denoted by *F'*.

Ratio in Experiment 72 (by APPEL).

D	0.2	0.4	0.6	0.8	1.0	1.2
F'	$^1/_{30}$	$^1/_{12}$	$^1/_{59}$	$^1/_{68}$	$^1/_{77}$	$^1/_{77}$

Ratio in Experiment 73 (by APPEL).

D	0.2	0.3	0.4	0.5	0.6	0.7	0.8
F'	$^1/_{32}$	$^1/_{48}$	$^1/_{52}$	$^1/_{79}$	$^1/_{80}$	$^1/_{97}$	$^1/_{103}$

Ratio in Experiment 74 (by VOLKMANN).

D	0.4	0.6	0.8	1.0	1.2	1.4
F'	$^1/_{26}$	$^1/_{48}$	$^1/_{43}$	$^1/_{47}$	$^1/_{44}$	$^1/_{44}$

Ratio in Experiment 75 (by VOLKMANN).

D	0.2	0.4	0.6	0.8	1.0	1.2	1.4
F'	$^1/_{19}$	$^1/_{42}$	$^1/_{55}$	$^1/_{63}$	$^1/_{68}$	$^1/_{68}$	$^1/_{73}$

Ratio in Experiment 76 (by JAHN).

D	0.3	0.5	0.7	0.9	1.1	1.3
F'	$^1/_{44}$	$^1/_{62}$	$^1/_{69}$	$^1/_{74}$	$^1/_{83}$	$^1/_{85}$

Ratio in Experiment 77 (by GEISS).

D	0.3	0.5	0.7	0.9	1.1	1.3
F'	$^1/_{122}$	$^1/_{188}$	$^1/_{256}$	$^1/_{333}$	$^1/_{345}$	$^1/_{345}$

Ratio in Experiment 78 (by KRAUSE).

D	0.3	0.5	0.7	0.9	1.1	1.3
F'	$^1/_{47}$	$^1/_{75}$	$^1/_{100}$	$^1/_{127}$	$^1/_{155}$	$^1/_{198}$

It follows from this compilation that the relative errors of equalization, and thus the smallest noticeable differences, increase with increasing smallness of the compared distances.

§69. To better justify the result of the just discussed experiments that were conducted by the *method of the mean error*, I conducted other experiments by the *method of the smallest noticeable differences*.

Experiment 79.

Performed by Mr. Krause by means of the screw micrometer at 200 millimeters viewing distance. The procedure is the following […]

p. 130

[Experiment 79, performed by stud. med. Krause]
[…]

Three distances were used, 0.5^{mm}, 0.9^{mm}, and 1.3^{mm}, and 80 experiments were performed with each, namely 40 with leftward and 40 with rightward location. Half of the 40 served for providing positive differences (= +u) and the other half negative (= –u). Full particulars are in the table.

Sum of the Differences for 20 Observations, Expressed in Millimeters.

	Distances					
	0.5 mm		0.9 mm		1.3 mm	
	—u	+u	—u	+u	—u	+u
L	0.274	0.296	0.275	0.279	0.279	0.313
R	0.280	0.293	0.254	0.297	0.306	0.256
Sum for 40 observations	0.554	0.589	0.529	0.576	0.585	0.569

Sum of the Plus- and Minus-Differences.

	0.5 mm	0.9 mm	1.3 mm
+u	0.589	0.576	0.569
—u	0.554	0.529	0.585
Sum for 80 observations	1.143 mm	1.105 mm	1.154 mm

[The first table summarizes the sums of differences for the 3×2×2 conditions, and the second table provides the sums of unsigned +u and –u conditions, which are 1.143 mm, 1.105 mm, and 1.154 mm for the 80 observations at 0.5 mm, 0.9 mm, and 1.3 mm, respectively. The sum of these three values (i.e. 3.402 mm) is then divided by the number of observations (240), giving 0.014175 mm. The inverse of that is 70.5, so the mean is 1/70 mm. The three individual differences are 0.0143 mm = 1/70 mm; 0.0138 mm = 1/72 mm; 0.0144 mm = 1/69 mm and are thus very similar. The following text, however, states the overall mean difference as 1/90 mm, and we believe that is a calculation error. As an aside, the correct value of 0.0142 mm corresponds to a visual angle of 14.6 seconds of arc at 200 mm viewing distance, which is again different from the 12.4'' given by Hering.]

It is thus revealed that the *absolute* value of the smallest discernible difference is the same for all distances, namely = 1/90 ^{mm} in the mean over 240 observations. This result deserves all the more confidence as it corresponds very well with that of Experiment 78 from the same observer [Krause].

p. 131
It remains to be said, how the smallest noticeable difference in KRAUSE's last experiment (79) relates to the respective distances [i.e. what the ratio is].

	Distances		
	0.5 mm	0.9 mm	1.3 mm
Relative Difference	$^1/_{35}$	$^1/_{66}$	$^1/_{90}$

[Because the absolute values of the smallest noticeable difference are the same, as said on the previous page, the three Weber fractions are different; the last of the three is $^{1}/_{90}$. By checking the calculations on the previous page it turns out that the absolute value for the three distances is, in fact, $^{1}/_{70}$ mm, not $^{1}/_{90}$ mm. It thus seems that Volkmann by mistake took the Weber fraction from the above table as the value for the smallest noticeable distance in mm. The calculation error was overlooked by Wundt (1874) and Hering (1899).]

p. 181

VI. On single vision with two eyes

p. 192: Exp. 99

p. 200: Fig. 22

p. 202: Exp. 100

[…]

p. 222: Exp. 121

[…]

p. 230, bottom: Fig. 23
§94. […] To ask further: Where in the matching lines are the matching points?

To answer this question for the horizontal dividing lines I use an apparatus whose essential characteristic is explained by the schematic figure given below.

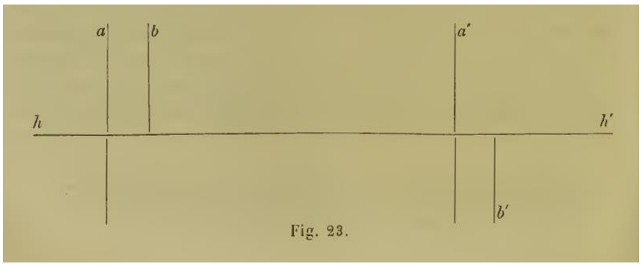

Fig. 23

p. 232

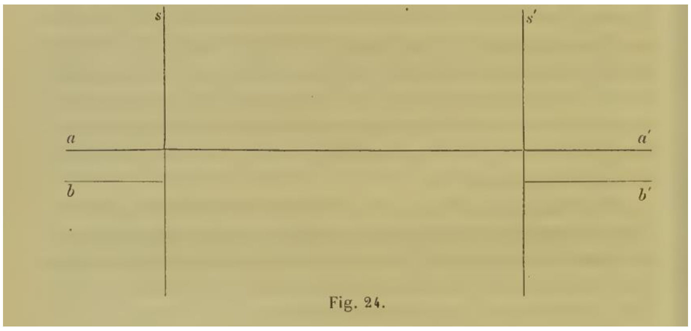

Fig. 24

p. 245 [bottom]
To exemplify what I said I will, in Fig. 28, denote the *fovea centralis* by *Aa*, and two corresponding retinal points on the horizontal meridian by *Bb*.

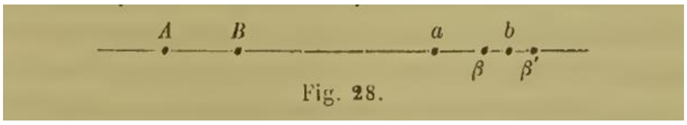

Fig. 28

When, by means of the instrument shown in Fig. 23, I seek for *B* in one eye the matching point (Deckpunkt) in the other eye, I will position the latter correctly to *b* in the mean of 30 experiments, but in many cases I will position it differently and, namely, in extreme cases to *ß* or *ß'*.

p. 246: occurrence of "Strecke"
ßß', or — what is the same — the difference *aß'–aß* thus denotes a range (Strecke), within which every point can serve a unitary sensation in binocular vision.

The difference of the maximal and minimal distance = *aß'–aß* was, in horizontal direction,

according to Exp. 129 = 5.55 – 4.75 = 0.80^{mm}

according to Exp. 130 = 5.55 – 4.85 = 0.70^{mm}

and in the perpendicular direction

according to Exp. 131 = 6.00 – 5.00 = 1.00^{mm}

according to Exp. 132 = 5.85 – 5.00 = 0.85^{mm}

in the mean of 120 experiments = 0.84^{mm}.

This difference causes, at the viewing distance of 300^{mm} that I used, a retinal image of 0.042^{mm}, which is 6-times larger than the smallest noticeable distance for my eye. In any case, the enormous size of the sensory circle (Empfindungskreis) found by Panum is to be considered a consequence of particularly unfavourable experimental conditions.

p. 267: Fig. 34

p. 268: End of Chpt. VI and end of book.
